# Production and Comprehension of Pantomimes Used to Depict Objects

**DOI:** 10.3389/fpsyg.2017.01095

**Published:** 2017-07-11

**Authors:** Karin van Nispen, W. Mieke. E. van de Sandt-Koenderman, Emiel Krahmer

**Affiliations:** ^1^Tilburg Center for Cognition and Communication, Department of Communication and Information Sciences, Tilburg University Tilburg, Netherlands; ^2^Rijndam Rehabilitation Center, RoNeRes Rotterdam, Netherlands; ^3^Erasmus Medical Center, Institute of Rehabilitation Medicine Rotterdam, Netherlands

**Keywords:** pantomime, idiosyncrasy, iconicity, individual variation, non-verbal communication, gesture

## Abstract

Pantomime, gesture in absence of speech, has no conventional meaning. Nevertheless, individuals seem to be able to produce pantomimes and derive meaning from pantomimes. A number of studies has addressed the use of co-speech gesture, but little is known on pantomime. Therefore, the question of how people construct and understand pantomimes arises in gesture research. To determine how people use pantomimes, we asked participants to depict a set of objects using pantomimes only. We annotated what representation techniques people produced. Furthermore, using judgment tasks, we assessed the pantomimes' comprehensibility. Analyses showed that similar techniques were used to depict objects across individuals. Objects with a *default* depiction method were better comprehended than objects for which there was no such *default*. More specifically, tools and objects depicted using a *handling* technique were better understood. The open-answer experiment showed low interpretation accuracy. Conversely, the forced-choice experiment showed ceiling effects. These results suggest that across individuals, similar strategies are deployed to produce pantomime, with the *handling* technique as the apparent preference. This might indicate that the production of pantomimes is based on mental representations which are intrinsically similar. Furthermore, pantomime conveys semantically rich, but ambiguous, information, and its interpretation is much dependent on context. This pantomime database is available online: https://dataverse.nl/dataset.xhtml?persistentId=hdl:10411/QZHO6M. This can be used as a baseline with which we can compare clinical groups.

## Introduction

When a train leaves from the platform, you can sometimes see people waving and gesturing at their loved ones. For instance, someone may form a heart shape with two hands, to depict their love for the other person. Such gestures, produced in absence of speech, are called pantomime (McNeill, 2000); throughout this paper, we will refer to these pantomimic gestures as “pantomimes.” Pantomimes may not be used as frequently as co-speech gestures, but their use can be convenient in situations in which speaking is not an option. Usually, it is assumed that the meaning of pantomime is not determined by any convention (McNeill, 1992). That is, the form and meaning of pantomimes does not meet any kind of socially constituted group standard. Instead, for the construction and comprehension of pantomime, people have to rely on iconicity, which is the similarity between the form and meaning of pantomime (Müller, 1998; Taub, 2001b; Perniss et al., 2010). Iconicity allows for a wide range of options to depict information in pantomime, but little is known about how people select from these options. Furthermore, although we know that pantomimes can convey information (McNeill, 2000), we know little of their comprehensibility. In general, very little is known about how people derive meaning from pantomime. The present study was initiated to investigate how people produce pantomimes and what kind of information others derive from pantomime. This can shed light on the question which mental processes people rely on for the production and comprehension of pantomime, something about which little is known.

Despite the uncertainties regarding how people construct and understand pantomime, in clinical settings, pantomime is often used as a clinical tool to support the communication of people with language difficulties, such as aphasia (e.g., Coelho, 1990; Raymer et al., 2006; Rodriguez et al., 2006; Daumüller and Goldenberg, 2010; Marangolo et al., 2010; Marshall et al., 2012; Caute et al., 2013). However, no clear baseline is available on how healthy speakers produce pantomime or on how comprehensible pantomime can be. This is an important gap in the extant literature. Therefore, the present study also aimed to provide a database to which clinical groups can be compared in future research.

### Pantomime on the gesture continuum

**Figure 1:**
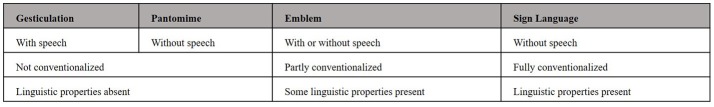
Kendon's continua: Relationship between gestures and speech, convention, and linguistic properties, as proposed by McNeill (2000).

McNeill, in his characterization of different kinds of hand gestures, placed pantomime on a continuum (Kendon's continuum as proposed by McNeill (2000), see Figure 1) in between gesticulation (i.e., gestures that spontaneously accompany speech), and emblems (i.e., gestures whose meaning is determined by conventions, such as the thumbs-up gesture). Pantomime, also sometimes called silent gesture, differs from gesticulation in that it is a conscious use of gesture in absence of speech (McNeill, 2000). Sandler (2013) defined pantomimes as reenactments of an event, in which the body represent the actual human body. In the present study, we take into account all gestures in absence of speech, but we focus on hand gestures only. Goldin-Meadow and Brentari (2015) propose a categorical divide between gesticulation and pantomime. Since pantomimes have a discrete form and can be concatenated into meaningful strings, pantomimes are more like signs than like co-speech gestures. The authors even propose to label pantomimes as “spontaneous sign.” It is worth noting, though, that sign language is generally considered to be very different from pantomime, in that it is a fully fledged language system with linguistic properties comparable to spoken language, such as a phonology, morphology and syntax (Emmorey and Casey, 2001; Sandler and Lillo-Martin, 2001). Rather than providing strict definitions, the gesture continuum is used by McNeill (1992, 2000) and McNeill and Duncan (1998) to illustrate that there can be a gradient transition between different gesture modes. Newly constructed pantomimes would be situated on the left side of the continuum, as for these pantomimes in principle no conventions exist. However, when used for a longer period of time within a certain community pantomime can take up linguistic properties and evolve toward signs in a sign language (e.g., Meissner et al., 1975; Coppola, 2002). In this case, pantomime will become better-formed, in that the hand shape and movement will increase in precision and will change in accordance with the grammar and rules of that evolving language (Singleton et al., 1993; Sandler et al., 2005, 2011; Brentari et al., 2012). Also, in speaking communities, in experimental settings, pantomimes that are used repeatedly are systematic in their order (Langus and Nespor, 2010; Hall et al., 2013, 2014). As a result of its frequent use in communication, the pantomimic depiction of a “telephone” has become an emblem in various cultures. In Italy for instance, people refer to the action of calling someone by holding a fist with stretched thumb near the ear and the little finger near the mouth, whereas individuals in America would hold a closed fist in a similar position between ear and mouth, but without the stretched thumb and little finger (Haviland, 2005).

### Iconicity

Although conventions may arise after frequent use of a pantomime, for the construction of new pantomimes, one cannot rely on conventions, or on linguistic rules, just yet. This raises the question of how people refer to concepts using new pantomimes, and how it is that others can generally understand these. To construct iconic gesture or new iconic signs, people likely rely on iconicity, which can be characterized as the similarity between (communicative or linguistic) form and a (real-world) referent or experience (Müller, 1998; Taub, 2001b; Perniss et al., 2010). This is probably also relied upon for the construction of pantomime. As Perniss and Vigliocco (2014) point out, iconicity maps form onto meaning, and thereby enables referring to things that are spatially and/or temporally remote. In this way, iconic pantomimes can also provide information for an interlocutor, just as iconic signs can convey information, though often ambiguous, to non-signers (Klima and Bellugi, 1979). This makes iconicity particularly useful for communication in situations in which no linguistic context is present.

The phone emblems discussed above are clearly iconic in that they represent the form of the telephone and the action of holding it. The Italian and American representations also show subtle differences, which illustrates that iconicity provides various options in the depiction of information in pantomime. A similar phenomenon is present in sign languages. Not only are there differences between sign languages in how they represent certain features of a concept, they can also differ in which feature of an object they express. In American Sign Language (ASL), for instance, a lion is represented by its salient feature “manes,” whereas in British Sign Language (BSL) it is represented by its pouncing paws (Perniss et al., 2010).

The gesture literature uses various labels to describe the manner of depiction people could use to express different types of information in pantomime (see e.g., Caldognetto and Poggi, 1995; Müller, 1998; Tolar et al., 2008; Cocks et al., 2013; Mol et al., 2013; Sekine and Rose, 2013; Hwang et al., 2014; Perniss and Vigliocco, 2014; Brentari et al., 2015). We based the present study on Müller (1998). She describes four modes of depiction: (1) the hand imitates the performance of everyday activity, (2) the hand molds, (3) the hand draws, and (4) the hand portrays an entity.

Considering the wide range of options that exist for depicting information in pantomime in an iconic fashion, one might expect substantial individual variation in how different people produce pantomimes. Only recently, this topic has gained more attention, and studies revealed systematic aspects in how people produce pantomime. Padden et al. (2013, 2015) showed that when depicting tools in pantomime, most people prefer to pretend to use the object, but some use their hands to represent the object. These findings were corroborated for co-speech gestures by Masson-Carro et al. (2016). In addition, findings by Hwang et al. (2014) indicate that people use specific strategies for different semantic categories; i.e., for animals, people use their hands to represent the animal, and for fruits, people show the shape. Furthermore, Brentari et al. (2015) found that people were more likely to depict agentives with the use of a handling techniques and non-agentives with an object technique when having to describe pictures without talking. This shows consistency in how people depict information in pantomime, which might indicate that these pantomimes are based on mental representations that are intrinsically similar (Barsalou, 1999).

### How do people produce pantomime?

The systematic aspects found in the production of pantomime suggest that different people use similar strategies when constructing pantomime. This raises the following question: how they do so in the absence of any conventions and what mental processes are involved? The underlying model of pantomime production is still poorly understood. Various models have been developed and tested to explain the production of co-speech gestures (see de Ruiter, 2000; Krauss et al., 2000; Kita and Özyürek, 2003). However, the production of pantomime is a process partly different from the production of co-speech gestures (Goldin-Meadow et al., 2008; van Nispen et al., 2014) and these models do not explain how iconic information is selected and translated into the manual domain. Even though no dedicated models of pantomime production exist, we feel that there are two models, developed for different purposes, which may serve as a source of inspiration.

One is the model of Gonzalez Rothi et al. (1997), which explains motor difficulties of people with apraxia. Apraxia is a disorder involving the performance of learned, purposeful movements (Gonzalez Rothi and Heilman, 1997). The model of Gonzales Rothi and colleagues describes which processes are involved in pretending to use an object, such as pretending to brush your teeth. According to this model, action semantics are selected from a semantic system or mental representation and these are subsequently translated into motor actions.

**Figure 2:**
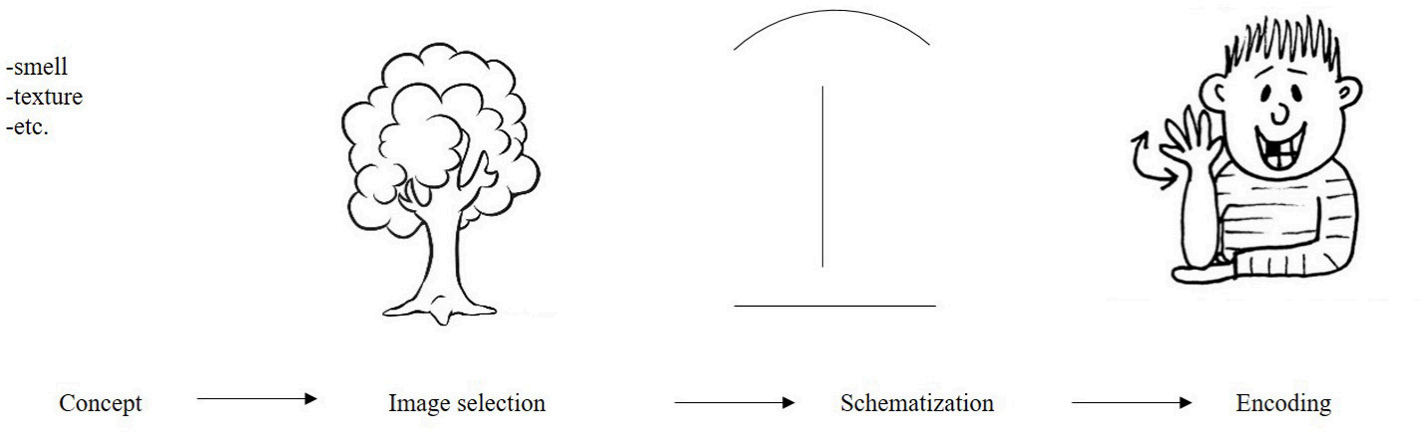
Analog-building process for American Sign Language (ASL) for the object tree as proposed by Taub (2001a).

Whereas the former model is well suited to explain specific disorders in the use of skilled movements, such as most action related pantomimes, it does not explain the creation of new pantomimes, such as outlining the shape of a toothbrush. The latter can be explained using the model by Taub (2001a). Inspired by the iconicity of some signs in sign language, she described how iconic items are created. Arguably, this model can also be used to explain the construction of pantomime. Taub (2001a) proposed that for the construction of iconicity, an image of an item is selected from one's mental representation, for instance an image of a tree (Figure 2). This image is created in the modality in which it will be represented, in this case, the visual domain. The (mental) image is then modified or schematized so that it can be depicted in a sign. From this schematized picture, one then selects appropriate forms or representable parts to show or encode (for instance, its vertical shape and branches). Taub (2001a) argued that whereas for the construction of linguistic items these are constrained by the semantic and phonetic categories of the language, (panto) mimes are constrained only by the conceptualizing power and physical skills of the person pantomiming.

The models by Gonzalez Rothi et al. (1997) and Taub (2001a) provide insight into how concepts can be depicted in various representation techniques. However, particularly the model by Taub (2001a) presumes individual differences in the construction of these pantomimes and does not explain the systematicity across individuals in the pantomime techniques produced for objects as reported in the literature (Padden et al., 2013, 2015; Masson-Carro et al., 2015, 2016). Therefore, we propose to further specify two selection criteria: saliency and fit with the constraints of the pantomime domain. These additional constraints may explain how people select the features they depict in pantomime.

As McRae et al. (2005) describe in their database of semantic object norms, there can be many features associated with an object; take the word “Whistle,” for example. These features can reflect a variety of basic knowledge types, such as information on its sound, shape and function (based on Wu and Barsalou, 2009). Following Taub's (2001a) model, a person producing a new pantomime will make a visual image of an object. This visual modality excludes all non-visual features, such as sound. Within the visual modality, other constraints attributed to the gesture domain remain. One can easily depict highly imageable content (Hadar and Butterworth, 1997) and particularly physical or spatial properties (e.g., Alibali, 2005), but other properties (for instance color, which also is a visual feature) may be more challenging. Consequently, for depicting an object in pantomime, people have to select a conceptual feature from their mental representation that meets the constraints of the pantomime domain, which are probably action (use of a toothbrush) or perception based (the shape of a funnel).

People may not depict all features depictable in pantomime. Rather, it seems plausible that people focus on salient, or distinctive information. Salient features are features that are remarkable and not shared with other objects (McRae et al., 2005). As such, salient features are most likely to be correctly understood by an interlocutor. However, it is important to note that salient information in the gesture domain may be different from salient information in other domains, such as the verbal domain. For instance, the feature “used to blow air through” may not be distinctive in language (since it applies to “Whistle,” but also to “Harmonica”), but in pantomime the differences in handshape in pretending to use the objects can be distinctive (“pretending to hold a whistle between thumb and forefinger” vs. “holding two hands with the palm up and arched fingers to pretend to hold a harmonica”).

Finally, as discussed above, there may be various ways in which a selected feature can be translated into a representation technique. It remains unclear how individuals select techniques. Taking into account that pretending to use an object is a depiction of a skilled action, something one has performed before, for which a motor program is readily available (Gonzalez Rothi et al., 1997; Hostetter and Alibali, 2008), it may be expected that this technique is preferred over the other techniques.

### How do people understand pantomime?

Given that there are no conventions on the production of pantomime, the question remains: how individuals can actually derive meaning from the pantomimes they observe? Although various studies have shown that gestures can be comprehensible (Kelly and Church, 1998; Beattie and Shovelton, 1999; Mol et al., 2013; Ping et al., 2014) and that iconic gestures activate semantically related information (e.g., Yap et al., 2013), very little is known about the cognitive processes involved in deriving meaning from pantomime. Therefore, we speculate on how this process might work and take Taub's (2001a) model as a starting point. If an interlocutor would see the iconic depiction of a tree, as shown in Figure 2, this person would need to deduce a scheme from this pantomime (a narrow, but somewhat long vertical shape, with a potentially moving wider top, and a flat base). This then needs to be translated into an image and linked to a concept (this could be a “tree,” “streetlight,” “flower,” “hat stand,” etc., …). While for linguistic items people have access to a lexicon providing clear links between form and meaning, for pantomime, this is obviously not the case. The scheme of this interlocutor does not have to map one on one onto a specific image or concept; what's more, it does not necessarily map onto the same concept as intended by the person producing the pantomime. Rather, the meaning of pantomimes is probably ambiguous and context dependent. Therefore, multiple interpretations could be possible. Two types of pantomimes might be less ambiguous: pantomimes that represent human action and pantomimes outlining or molding a salient shape of an object. For most pantomimes, the person producing the pantomime and the addressee will not share the same way of schematizing, which can result in an ambiguous message. For human action, however, the schematization is shared between interlocutor and pantomimer, because this is skilled action, previously performed by both individuals. An interlocutor can understand, for instance, a pantomime where an individual pretends to “comb his hair,” as the interlocutor can map this action onto own experience. For pantomimes molding the shape of an object, interlocutors cannot map the scheme of a pantomime onto their own experience. That said, a shape might be recognized similarly to how the shape of an object is recognized in, for instance, a line drawing (e.g., Biederman, 1987). This will probably only work for objects with a salient shape, that are recognizable based on their shape only, such as for instance a “Pyramid,” but not for objects with ambiguous shapes, such as a “Bed.”

### Who needs pantomime?

As language is a very efficient communication system, most of the time, there is no need for people to use pantomime. There are some situations in which pantomime could be a useful alternative: when speakers do not share a language (for instance, when traveling abroad) or when communication using sound is difficult or impossible (for instance, at a train station when a train passes by or when trying to communicate through a glass window). To what extent people actually rely on pantomime in such situations is largely unknown.

For some clinical populations, particularly for individuals with language difficulties, pantomime could be useful for communicating information they cannot (or can no longer) convey in speech. People with aphasia (Goodglass, 1993) and children with Specific Language Impairment, SLI (Leonard, 2014) have linguistic impairments, but also children with Down syndrome (Chapman and Hesketh, 2000) may struggle with the production of language. Some studies have shown that these populations can sometimes use gesture or pantomime to convey information they cannot convey in speech, which may improve their comprehensibility to others (Stefanini et al., 2007; Botting et al., 2010; Mol et al., 2013). Due to the possible benefits of pantomime for these clinical populations, there is a need to collect data on the ability of healthy speakers to use pantomime. A baseline database for pantomime can be used to identify what works well in healthy speakers and should be encouraged in clinical populations. Furthermore, it may be used to define the “best possible outcome” which can be used to guide expectations in clinical populations. It can also identify caveats which can be avoided with clinical populations. Finally, a baseline could be used to determine pantomime impairments. For instance, people with apraxia have difficulties performing skilled movements, such as pretending to brush one's teeth (Gonzalez Rothi and Heilman, 1997). Also, people with aphasia seem to use pantomime differently from healthy participants (van Nispen et al., 2016). A pantomime baseline could be used as a comparison to the behavior of clinical groups in order to determine which aspects of pantomime production they struggle with.

### Current study

#### Aim

We aimed to investigate how (healthy) people produce and comprehend pantomimes. This topic has received little attention, with the notable exception of the aforementioned study by Padden et al. (2015). The present study determined which representation techniques people used to depict objects from the Boston Naming Test, BNT (Kaplan et al., 1983), a standardized test used to assess naming impairments in people with aphasia. Furthermore, we looked into whether there were systematic aspects to how these techniques were applied. Using two judgment tasks, open question and forced-choice, we determined the comprehensibility of these pantomimes. Our study is based on three hypotheses. Firstly, considering the communicative use of pantomime we expected to find systematic aspects in how people depict objects in pantomime. Based on the regularities observed by Padden et al. (2015) we expect to find that across individuals similar techniques are used for certain (categories of) items. Secondly, considering the constraints of the pantomime domain, we hypothesized that pantomime is best used to depict objects with a salient function, particularly tools, and that these items would be better understood than items without a salient function. Finally, because of the iconic information present, but the lack of conventions regarding its meaning, we expected pantomimes to convey information that is semantically rich, but ambiguous and therefore highly context dependent. Interpretation of a pantomime is not based on conventions, but depends on the interpretation of the interlocutor and the context is which it is produced. Based on Gonzalez Rothi et al. (1997) and Hostetter and Alibali (2008), we also hypothesized that pantomimes depicting objects using a *handling* technique might be easier to understand, since these can be mapped onto a motor program shared with the interlocutor, which increases the likelihood of the correct interpretation of the pantomime.

#### Pantomime database

The second aim of this study was to build a pantomime database. As alluded to above, in clinical settings, pantomime is sometimes used as a manner of communication for people with language difficulties, such as aphasia (e.g., Coelho, 1990; Raymer et al., 2006; Rodriguez et al., 2006; Daumüller and Goldenberg, 2010; Marangolo et al., 2010; Marshall et al., 2012; Caute et al., 2013). No reliable information is currently available on how healthy individuals produce and understand pantomime. Therefore, the present study also aimed to provide a database to which clinical groups can be compared.

## Pantomime elicitation

### Participants

Twenty native speakers of Dutch participated in the experiment (5 male), aged 32–65 (*M* = 53). They were all right handed, as assessed using the Edinburgh Handedness Inventory (Oldfield, 1971). Participants gave their consent to be videotaped during the experiment.

### Stimuli

Stimuli were all 60 pictures of the Boston Naming Test, BNT (Kaplan et al., 1983). The pictures in this test depict various objects, animals and plants (from now on referred to as objects) which increase in naming difficulty, from high frequency words, such as “House,” to low frequency words, such as “Compass.” This test was selected for its clinical relevance, as it was also used with people with aphasia for a different study (van Nispen et al., 2016).

### Procedure

Participants saw a picture and were asked to silently convey what was on that picture by using only their hands, i.e., by pantomiming. This had to be done in such a way that the experimenter, who could not see this picture because of a cardboard screen, could select the correct picture from three answer options. Before starting the task, three practice items were used to familiarize the participants with the task. After participants had completed their pantomime for a practice item, the experimenter showed the three answer options. She always indicated she had understood the pantomime by pointing to the correct answer. Before starting the experiment itself, participants were reminded that they should pantomime until they thought the information was clear enough to the experimenter and that said experimenter was not allowed to give feedback on the comprehensibility of the pantomime. During the experiment, answer options were not shown to the participants, nor did the experimenter give any feedback on the comprehensibility of a pantomime.

Due to minor mistakes in the test procedure, such as skipping a page unseen, there were two missing items (item 27 and 37 by participant 10). Analyses were performed for the remaining 1198 items (20 participants^*^60 items = 1200 − 2 missing items = 1198 items).

As this experiment was part of a larger research project also including stroke patients with aphasia, half of the participants were restricted in the use of their right hand. These randomly selected participants had to wear a sling throughout the experiment. This was done to make sure that the healthy speakers were comparable to the aphasic speakers in the database, of whom many had a right-sided hemiparesis (van Nispen et al., 2016).

The pantomimes produced were analyzed in two ways. Study 1 describes the representation techniques people used and Study 2 reports on how comprehensible these pantomimes were.

## Study 1. assessment of representation techniques

### Materials and methods

#### Coding

For each object, the pantomimes produced were annotated into different representation techniques using the ELAN gesture coding software package (Wittenburg et al., 2006). Basing our coding on Müller (1998), we identified six representation techniques (also used in Mol et al., 2013; van Nispen et al., 2014, 2016). Within Müller's category of gestures imitating daily activity, we distinguished between: (1) a *handling* technique, which is a transitive action, in which one pretends to use an object (e.g., pretending to hold a whistle), and (2) enacting, which is an intransitive action, or non-object-directed action (e.g., pretending to be dancing or swimming). We combined Müller's mold and draw modes of representation and labeled this as (3) *shape* (e.g., outlining or molding the shape of a whistle). We labeled the “portraying” techniques as (4) *object*, in which the hand represents the object (e.g., use fingers to represent a whistle). In addition to Müller's modes of representation, we also distinguished (5) *deictic* (e.g., pointing at one's mouth) and (6) *other*, which were all pantomimes that did not fit into previous categories, see Table 1. Coding was done by the first author. Second coding was done for 10% of the items by two different coders, both experts in gesture coding, who each coded part of this 10%. For every item we determined whether or not a technique was used. For instance, someone could pretend to brush their teeth and pretend to put toothpaste on their toothbrush, these are both handling techniques, therefore only one technique is noted for this item. This method does not allow for calculating kappa, as there were some instances for which one coder identified more techniques than the other. We report agreement instead, which was 76% for the identified techniques. It is important to note that for the codes of interest, agreement was particularly high; Out of the 564 handling techniques identified by the first coder in this sample, the two coders agreed on 93%. For the *object* technique agreement was 78% (*N* = 415) and for *shape* 86% (*N* = 712). Only for the techniques that were used infrequently agreement was lower: enact (59%) deictic (67%) and other (45%).

**Table 1:** Coding scheme for representation techniques used (van Nispen et al., 2014, 2016).

**Representation technique**	**Definition**	**Example**
*Handling*	Pretending to use an object	Pretending to write with a pencil
*Enact*	One pretends to be in a different situation, without using an object	Pretending to be cold by rubbing one's hands to opposite shoulders
*Object*	Using one's hands to represent (part of) an object	Holding a hand in front of one's face for representing a mask
*Shape*	Outlining or molding the shape of an object	Drawing the outline of a house with one's index finger
*Deictic*	Pointing (index finger) at object, location or trajectory	Pointing at one's chair
*Other*	All gestures that do not fit into previously named categories	Showing three fingers for representing the number “three”

#### Analyses

For the analyses, we determined for each item whether or not one of the six techniques was used. This means that a technique could be used maximally 60 times (once for every item) by every individual and that multiple techniques could be used to depict a single item. We performed three types of analyses. First, as half of our participants wore a sling during the experiment, we performed an independent samples *t*-test to check whether people restricted in the use of their right hand differed from people able to use both hands. Secondly, we set a threshold: if 80% or more (≥16/20) of the participants used the same technique for a specific object we labeled this as a *default* technique. Thirdly, based on Padden and colleagues (Hwang et al., 2014; Padden et al., 2015), we determined differences in the representation techniques used to depict animals (*n* = 8), tools (*n* = 16) and other (*n* = 36). Tools were categorized as a handheld device that aids in accomplishing a task. Groups of items were compared using a MANOVA with Bonferroni's *post-hoc* testing.

### Results

See **Table 3** for an overview of the techniques used per item. Participants on average used 1.77 (*SD* = 0.38) techniques per item. They used a shape technique for most items (*M* = 0.59, *SD* = 0.17), handling for about half of the items (*M* = 0.47, *SD* = 0.06), object (*M* = 0.35, *SD* = 0.11), and enact (*M* = 0.13, *SD* = 0.10) were used to a lesser degree.

#### Restricted hand use

There were no significant differences between people able to use only one hand and people able to use both hands for the use of any of the representation techniques: [*handling t*_(18)_ = 0.38, *p* = 0.707, *object t*_(18)_ = − 0.95, *p* = 0.357, *enact t*_(18)_ = −0.61, *p* = 0.551, *shape t*_(18)_ = −0.79, *p* = 0.438, *deictic t*_(18)_ = −0.79, *p* = 0.443, and *other t*_(18)_ = −0.57, *p* = 0.576]. Therefore, for the following analyses, the data of both participant groups were collapsed.

#### Defaults

**Figure 3:**
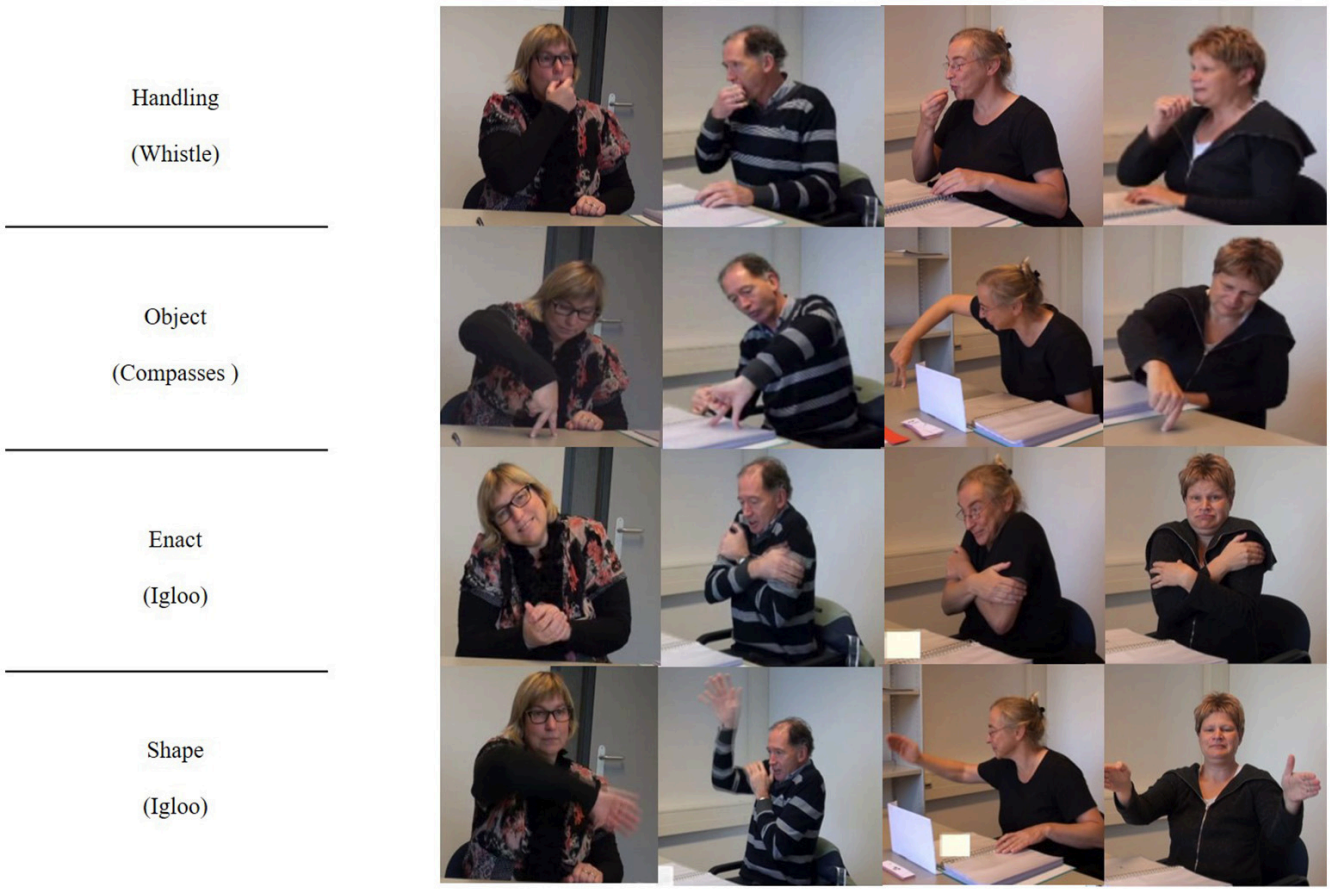
Participants using a *default* pantomime technique for the objects “Whistle,” “Compass,” and “Igloo.”

For 52 out of 60 objects (87%), a *default* technique was used, i.e., 80% or more of the participants used a certain technique in their depiction of that object, see Figure 3 for some examples. This confirmed that there are systematic aspects to the way people refer to objects in pantomime. Note that objects could have one, two, or even three *default* techniques. *Handling* was the *default* technique for 19 objects, *enact* for 2 objects, *object* for 10 objects and s*hape* for 24 objects (Table 2). For 4 objects, people used either a *handling* or an *object* technique. Both techniques reflected the same information: use of an object (e.g., for “Saw”: pretending to hold a saw and move it back and forth or showing a flat hand perpendicular to the table and move it back and forth). For 46 out of 60 objects, people used a single technique as *default*. For 5 objects, two techniques met the threshold of 80% or more. These *default*s were always combinations of *shape* plus another technique. For “Cactus,” three techniques were *default: shape* (molding/outlining the shape of the cactus), *handling* (pretending to touch a thorn of the cactus) and *enact* (pretending to be hurt, by shaking the hand). In addition to the above named *default* techniques, individuals sometimes added other representation techniques in their depiction of an object. Those techniques, though, were not used by 80% or more of the participants and are thus not reported here.

**Table 2:** Objects per *default* technique used by percentage of participants (The number between brackets indicates the order of the items in the Boston Naming Test. A higher number is indicative of lower imageability and word frequency).

***Handling***	***%***	***Object***	***%***	***Shape***	***%***	***Enact***	***%***
Accordion [47]	100	Helicopter [11]	100	Globe [27]	100	Igloo [33]	90
Broom [12]	100	Bed [1]	90	Trellis [57]	100	Cactus [36]	80
Dart [25]	100	Compass [50]	90	Pyramid [43]	100		
Harp [38]	100	Muzzle [44]	90	Acorn [32]	95		
Pallet [58]	100	Pelican [41]	90	Camel [17]	95		
Pencil [3]	100	Snail [22]	90	Funnel [46]	95		
Racquet [21]	100	Volcano [23]	85	Igloo [33]	95		
Comb [7]	95	Mask [18]	80	Mushroom [14]	95		
Knocker [40]	95	Octopus [13]	80	Rhinoceros [31]	95		
Harmonica [30]	95	Sphinx [55]	80	Cactus [36]	90		
Scroll [53]	95			Unicorn [45]	90		
Stethoscope [42]	95			Wreath [28]	90		
Toothbrush [10]	95			Abacus [60]	85		
Wheelchair [16]	95			Asparagus [49]	85		
Whistle [5]	95			Bench [20]	85		
Abacus [60]	90			Noose [48]	85		
Canoe [26]	90			House [4]	85		
Latch [51]	90			Protractor [59]	85		
Cactus [36]	85			Snail [22]	85		
Either/Or^*^				Tripod [52]	85		
Noose [48]	50		45	Yoke [56]	85		
Saw [9]	65		40	Hanger [15]	80		
Scissors [6]	35		70	Pelican [41]	80		
Tongs [54]	55		65	Tree [2]	80		

* *Either/or were all objects that were depicted by >80% of participants by either a handling (between 35 and 80%) or an object technique (between 35 and 80%)*.

#### Object classifications

**Figure 4:**
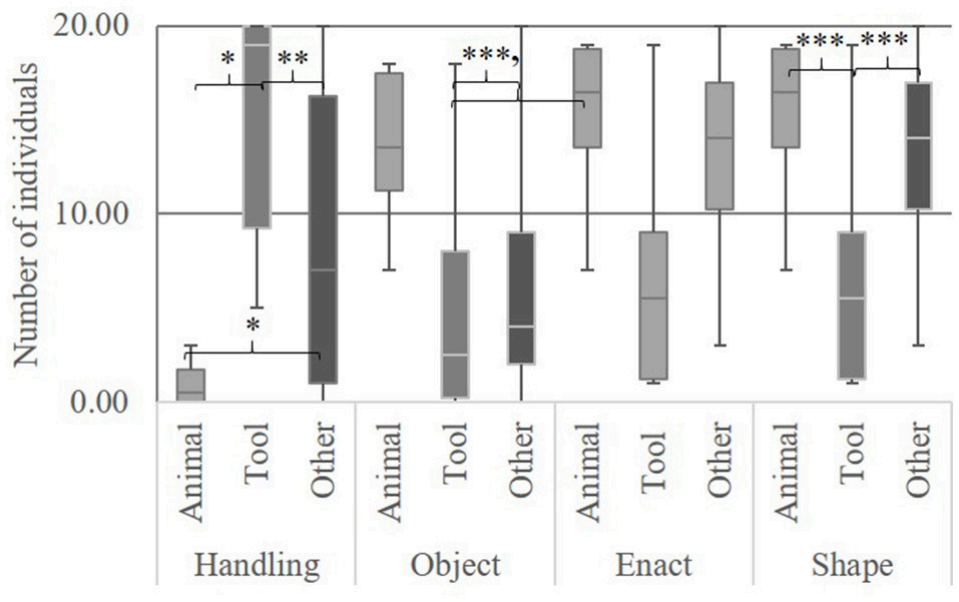
Representation techniques used by number of individuals for tools, animals and other. Error bars show *SD* '*p* < 0.070, ^*^p < 0.050, ^**^p < 0.010, ^***^p < 0.001.

We found differences between objects, tools and other in the degree to which they were depicted with a *handling* technique, *F*_(2, 56)_ = 14.73, *p* < 0.001, object techniques, *F*_(2, 56)_ = 6.74, *p* < 0.002, and *shape, F*_(2, 56)_ = 10.11, *p* < 0.001 (Figure 4). No difference was found for *enact, F*_(2, 56)_ = 2.38, *p* = 0.102. Bonferroni's *post-hoc* testing revealed that tools were depicted significantly more often by a *handling* technique than animals (*M*_*diff*_ 14.69, *p* < 0.001) and other (*M*_*diff*_ 7.01, *p* = 0.002). Other were depicted more often by a *handling* technique than animals (*M*_*diff*_ 7.68, *p* = 0.010. Animals were depicted more often by an *object* technique than tools (*M*_*diff*_ 8.88, *p* < 0.002) and other (*M*_*diff*_ 7.24, *p* < 0.006). *Shape* was used more often for animals (*M*_*diff*_ 8.63, *p* < 0.001) and other (*M*_*diff*_ 6.40, *p* < 0.001) than for tools.

## Study 2: assessment of pantomime's comprehensibility

### Materials and methods

#### Judges

To assess the comprehensibility of the pantomimes, we included 273 Judges in our study. These were all students of either Communication and Information Sciences at Tilburg University, or Speech Language Pathology at Hogeschool Rotterdam (age *M* = 21, *SD* = 4). There were 152 judges performing open question judgements and 121 for the forced-choice experiment. They were all naïve to the purpose of this study.

#### Materials

The materials consisted of videos of each participant described in the pantomime elicitation. These videos were cut into clips per item. A clip started when the participant had turned the page to that item and ended when the participant moved his hand to the page to go on to the next item. This resulted in 1198 clips (60 objects^*^20 participants − 2 missing items).

#### Comprehensibility assessment

In both experiments, each clip (depicting all pantomimes that were produced by a participant for a certain item) was seen by three judges. This resulted in 7,194 judgments (= 2 experiments^*^1198 clips^*^3 judgments). For each clip, judges had to answer the following question; “Watch the video above. What is this person depicting?” For the open questions the following was added: “Note, it is always an object, animal or plant.” The forced-choice experiment, answer options were one correct answer and three distracters, all randomly selected from the other pictures in the BNT. These distracters were always the same for a certain object, but their order on the screen was varied between judges and participants producing the pantomimes.

#### Analyses

Due to technical problems, there were two missing items (participant 1 item 3 and particpant 10 item 1) in the open question experiment and 2 missing items (2^*^participant 1 item 2) in the closed answer experiment. Analyses were performed on the remaining items.

Comprehensibility of the gestures was operationalized in three scores: (a) Correct score forced-choice: an average Correct score for the forced-choice questions, (b) Correct score open: an average Correct score for the open questions, based on the correct responses described in the Dutch manual of the BNT (van Loon-Vervoorn et al., 1996) and (c) Semantic score open.

Responses from the different questions were transformed into these scores as follows: For the correct score forced-choice, the score was based on whether the three judges had identified an item correctly. Therefore, for each individual, an item could be 0, 33, 67, or 100% correct (no judge, one judge, two judges, or all three judges identified the item correctly). For the analyses per individual we calculated an average correct score over the 60 items they depicted. For the other analyses, over items, we calculated the correct score per item, an average over the 20 participants. The transformation of the Open-ended questions was performed similarly, only now, the correct identification of a pantomime was based on the correct responses described in the Dutch manual of the BNT (van Loon-Vervoorn et al., 1996). The semantic score is a four point scale in which semantic similarities are taken into account. Scores were given following the guidelines of van Loon-Vervoorn et al. (1996). For instance “Pen” is not the correct answer for the object “Pencil,” but it is semantically closely related and would be scored with a 2 (on a scale from 0 to 3). This score was added since we expected gestures to be unspecific, but carrying ambiguous meaning that may convey semantically relevant information. See Appendix Table A1 for a detailed description of this scoring scale.

Similarly to Study 1, we performed three types of analyses. Firstly, using an independent samples *t*-test, we checked whether the comprehensibility of pantomimes created by people restricted in using their right hand as compared to people using both hands was relatively equal. Secondly, using an ANCOVA, we compared the comprehensibility of objects with a *default* technique to objects for which there is no such *default*. Since our perception study is an indirect measure of comprehensibility, the comprehensibility of a pantomime can be influenced by the judges' ability to identify and name the object. To control for this, we added “nameability” as a covariate for the analyses with the Correct score open and Semantic score open. Nameability is based on the Dutch norms of the BNT, and represents the degree to which healthy speakers are able to verbally name a picture of this object (van Loon-Vervoorn, 1985). Subsequently, we performed an ANCOVA with Bonferroni's *post-hoc* testing with nameability as a covariate when comparing the comprehensibility between the different *default* techniques. Finally, we looked into whether different classifications of the objects could explain the comprehensibility of the pantomimes. Using an ANCOVA with nameability as a covariate and Bonferroni's *post-hoc* testing, we compared tools (*n* = 16), animals (*n* = 8), and other (*n* = 36).

### Results

Table 3 shows the average comprehensibility scores per item and the proportion of participants who used a certain technique for this item.

**Table 3:** Representation techniques used by proportion of participants and comprehenisbily scores (averaged over participants) per item.

**Nr**	**Item**	**Technique used (by proportion of participants)**				**Comprehensibility scores (average over participants)**		
		***Handling***	***Object***	***Enact***	***Shape***	**Correct forced-choice**	**Correct open**	**Semantic open**
1	Bed	0.05	0.90	0.35	0.20	0.95	0.68	2.20
2	Tree	0.05	0.30	0.05	0.80	0.83	0.20	0.75
3	Pencil	0.40	0.25	0.05	0.85	0.88	0.03	1.44
4	House	0.50	0.15	0.20	0.70	0.87	0.37	1.13
5	Whistle	0.00	1.00	0.05	0.15	0.97	0.68	2.12
6	Scissors	0.30	0.10	0.00	0.95	1.00	0.92	2.80
7	Comb	0.65	0.05	0.00	0.80	1.00	0.58	2.43
8	Flower	0.95	0.20	0.05	0.25	0.85	0.17	0.98
9	Saw	0.20	0.80	0.00	0.60	1.00	0.67	2.07
10	Toothbrush	0.65	0.15	0.05	0.70	1.00	0.87	2.78
11	Helicopter	0.00	0.00	0.40	0.85	0.98	0.28	0.98
12	Broom	0.00	0.85	0.15	0.65	0.98	0.23	0.80
13	Octopus	0.85	0.20	0.10	0.55	0.92	0.07	0.38
14	Mushroom	0.25	0.15	0.00	1.00	0.90	0.13	0.40
15	Hanger	0.30	0.10	0.35	0.90	0.93	0.20	0.57
16	Wheelchair	0.95	0.00	0.00	0.25	0.90	0.50	1.50
17	Camel	0.20	0.10	0.00	0.95	0.90	0.17	0.50
18	Mask	0.00	0.35	0.90	0.95	1.00	0.48	1.48
19	Pretzel	0.70	0.10	0.10	0.65	0.90	0.12	0.60
20	Bench	0.55	0.10	0.05	0.75	0.93	0.17	0.83
21	Racquet	0.85	0.05	0.80	0.90	1.00	0.58	2.00
22	Snail	0.05	0.75	0.15	0.30	0.97	0.13	0.50
23	Volcano	1.00	0.00	0.05	0.55	0.97	0.08	0.47
24	Seahorse	0.15	0.45	0.35	0.65	0.85	0.07	0.33
25	Dart	0.95	0.10	0.00	0.45	1.00	0.40	1.57
26	Canoe	0.00	0.10	0.20	1.00	1.00	0.05	0.83
27	Globe	0.05	0.90	0.15	0.50	1.00	0.03	0.22
28	Wreath	1.00	0.00	0.15	0.20	0.77	0.02	0.05
29	Beaver	0.50	0.45	0.35	0.85	0.98	0.07	0.37
30	Harmonica	0.70	0.15	0.15	0.85	0.98	0.75	2.33
31	Rhinoceros	0.90	0.35	0.00	0.55	0.93	0.22	0.93
32	Acorn	0.50	0.30	0.00	0.85	0.95	0.00	0.00
33	Igloo	0.95	0.25	0.40	0.30	0.97	0.40	1.27
34	Stilths	0.00	0.80	0.15	0.60	1.00	0.03	0.18
35	Dominoes	0.15	0.45	0.15	0.85	0.98	0.07	0.25
36	Cactus	0.10	0.55	0.00	1.00	0.92	0.20	0.68
37	Escalator	0.00	0.80	0.20	0.35	1.00	0.12	1.07
38	Harp	0.00	0.35	0.15	0.95	1.00	0.55	1.68
39	Hammock	0.05	0.90	0.05	0.85	0.97	0.32	1.20
40	Knocker	0.10	0.60	0.15	0.75	0.97	0.03	0.88
41	Pelican	0.15	0.60	0.30	0.65	0.93	0.22	1.42
42	Stethoscope	0.00	0.55	0.05	0.95	0.93	0.20	0.72
43	Pyramid	0.00	0.90	0.10	0.80	0.98	0.30	0.92
44	Muzzle	0.05	0.75	0.10	0.90	0.98	0.07	0.32
45	Unicorn	1.00	0.00	0.05	0.20	0.95	0.20	0.70
46	Funnel	0.95	0.05	0.10	0.05	0.97	0.25	0.93
47	Accordion	0.35	0.70	0.00	0.05	1.00	0.33	1.20
48	Noose	0.95	0.10	0.05	0.10	0.95	0.50	2.00
49	Asparagus	0.65	0.40	0.00	0.05	0.85	0.00	0.00
50	Compass	0.95	0.15	0.00	0.05	1.00	0.43	1.32
51	Latch	1.00	0.10	0.05	0.10	0.95	0.03	0.35
52	Tripod	1.00	0.00	0.00	0.25	0.87	0.25	0.93
53	Scroll	1.00	0.00	0.05	0.40	0.97	0.05	0.30
54	Tongs	0.95	0.15	0.30	0.45	0.93	0.00	0.25
55	Sphinx	0.70	0.15	0.00	0.95	0.80	0.03	0.20
56	Yoke	0.25	0.90	0.00	0.30	0.73	0.00	0.00
57	Trellis	0.40	0.65	0.20	0.40	0.98	0.00	0.13
58	Palette	1.00	0.05	0.00	0.45	1.00	0.07	0.67
59	Protractor	0.40	0.40	0.00	0.85	0.93	0.00	0.12
60	Abacus	0.90	0.00	0.00	0.85	0.97	0.12	0.42

#### Restricted hand use

Pantomimes produced by people able to use both hands (*M* = 0.97, *SD* = 0.02) were slightly better comprehended than pantomimes produced by people able to use only their left hand (*M* = 0.93, *SD* = 0.03) for the Correct score forced-choice: *t*_(18)_ = −4.13, *p* <0.01, for the Correct score open (2 hands: *M* = 0.29, *SD* = 0.06 and 1 hand: *M* = 0.21, *SD* = 0.06): *t*_(18)_ = −3.15, *p* <0.01, and for the Semantic score open (2 hands: *M* = 1.10, *SD* = 0.13 and 1 hand: *M* = 0.81, *SD* = 0.18): *t*_(18)_ = −4.07, *p* <0.001. These differences were only minor and were not of interest to the scope of the present study. This issue is discussed in more detail in the discussion. For the following analyses we collapsed the data.

#### Default

**Figure 5:**
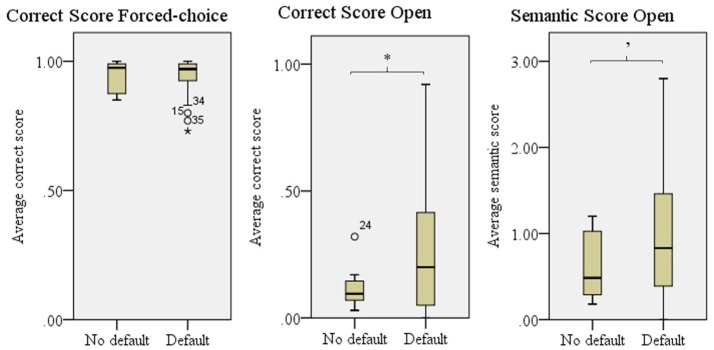
Comprehensibility scores for objects with a *default* technique and no *default* technique. Error bars show *SD*. '*p* = 0.070, ^*^p < 0.050.

Figure 5 shows that objects with no *default* had a lower Correct score in the open experiment than objects depicted with a *default* technique, *F*_(1, 57)_ = 4.74, *p* = 0.034. For the Semantic score open, we found a trend, *F*_(1, 57)_ = 3.43, *p* = 0.069. We found no differences for the Correct score in the forced-choice experiment, which is probably due to a ceiling effect.

**Figure 6:**
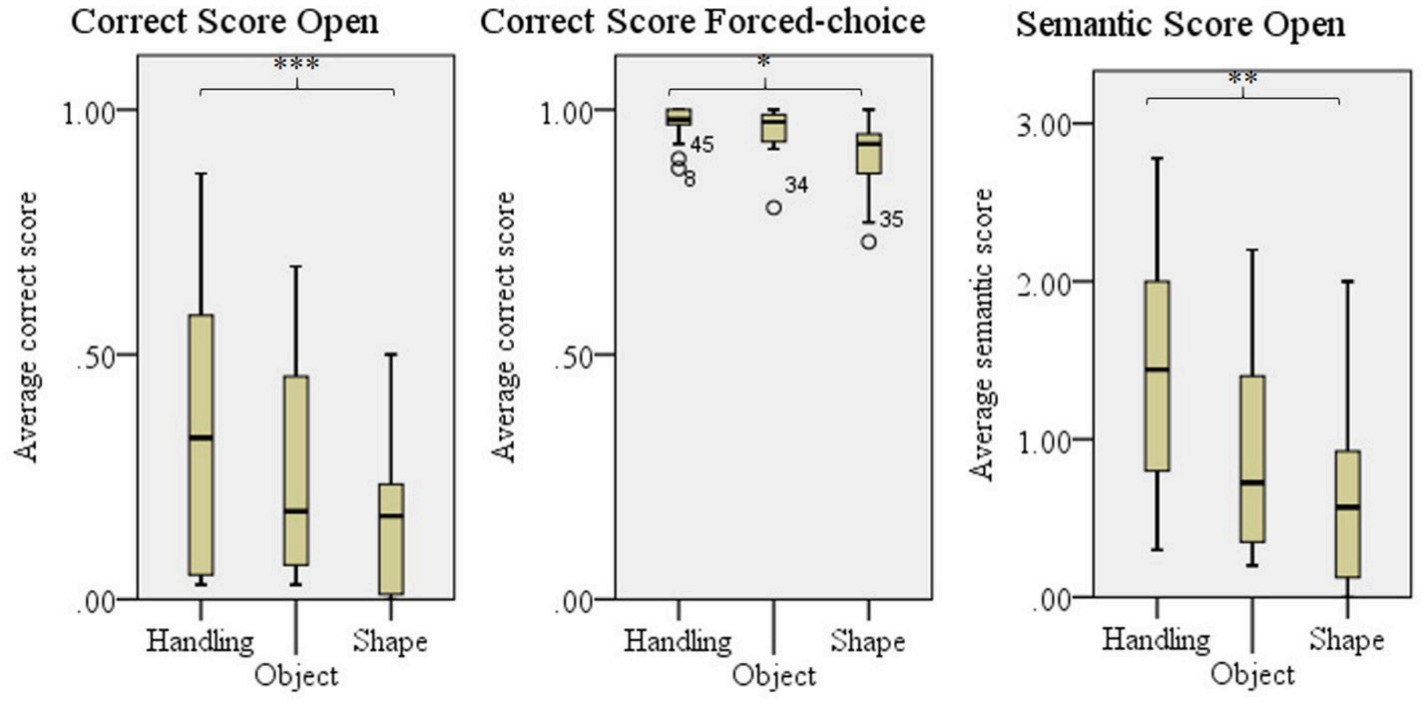
Comprehensibility scores for objects per *default* technique; Handling, Object, and Shape (used by 80% or more of the participants). Enact was not used frequently enough to perform further analyses on. Error bars show *SD*. ^*^p < 0.050, ^**^p < 0.010, ^***^p < 0.001.

The type of technique used as *default* influenced the comprehensibility as given in the Semantic score for the open experiment, *F*_(2, 41)_ = 6.84, *p* = 0.003 and Correct score in the forced-choice experiment, *F*_(2, 41)_ = 5.64, *p* = 0.007. For the correct score in the open experiment the difference was close to significance, *F*_(2, 41)_ = 3.20, *p* = 0.051. Bonferroni's *post-hoc* testing revealed that objects for which *handling* was the *default* technique were better comprehended than objects for which a *shape* technique was used as *default* in all three scores, see Figure 6. Since *enact* was used as a *default* for only two objects, this technique was not taken into account in these analyses.

#### Object classification

**Figure 7:**
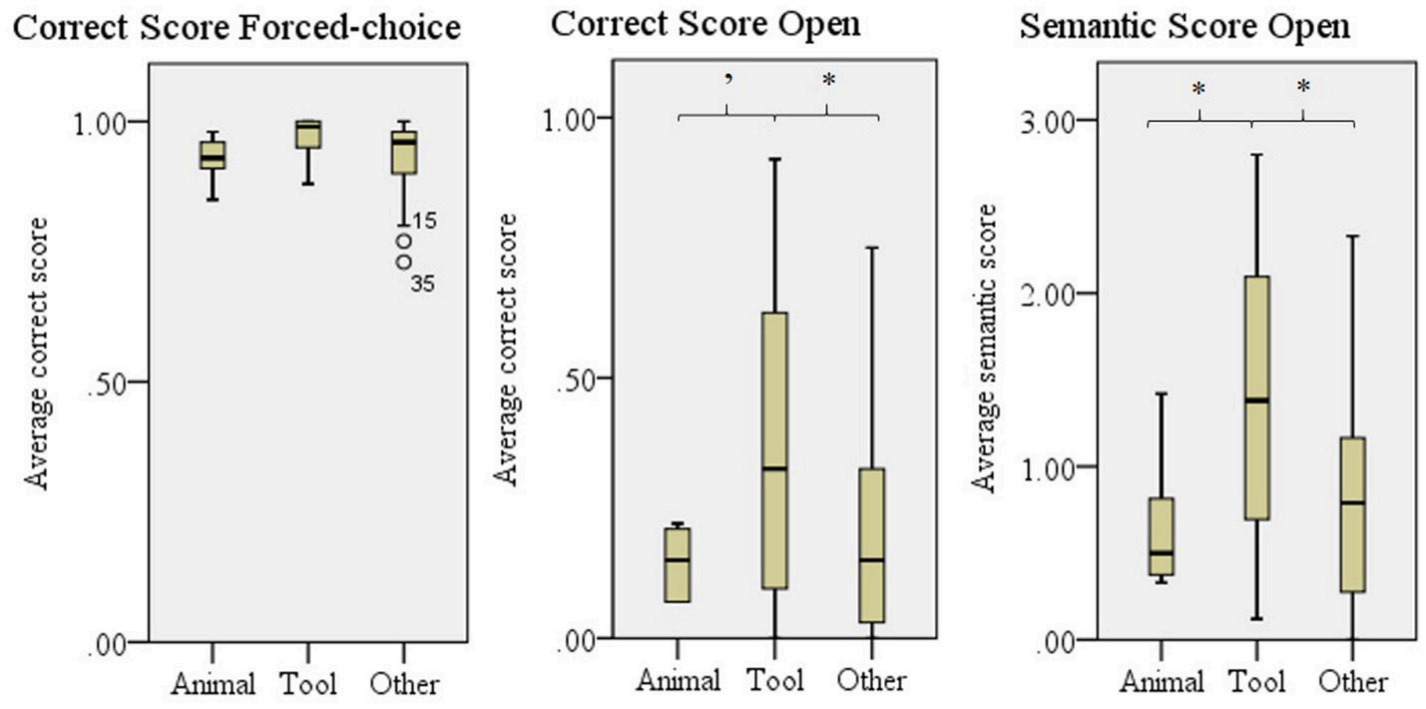
Comprehensibility scores for animals, tools and other. Error bars show *SD*. '*p* = 0.070, ^*^p < 0.050.

We found differences between the three categories for the Correct score open, *F*_(2, 57)_ = 3.95, *p* = 0.025, the Semantic Score Open, *F*_(2, 57)_ = 5.20, *p* = 0.008, and a trend for the Correct score forced-choice, *F*_(2, 57)_ = 2.67, *p* = 0.078 (Figure 7). Tools were better understood than both animals and other in both of the Open answer scores, but *post-hoc* testing revealed no significant differences for the forced-choice Correct score.

## Discussion

This study set out to investigate how people produce and comprehend pantomimes and whether there were systematic aspects in the manner in which objects were depicted. To this end, we determined which representation techniques participants used to depict a series of objects and we assessed the comprehensibility of these pantomimes. There were three major findings. Firstly, we found these systematic aspects, in that the same technique was used across individuals to depict a certain item, which suggests that pantomime is not fully idiosyncratic. Secondly, we found that tools were most often depicted by a *handling* technique, and animals most often by an *object* technique. Furthermore, tools were better comprehended than animals or other objects. This relates to our other finding that objects depicted by a *handling* technique were better understood than objects depicted by one of the other techniques. Finally, the meaning of pantomime is semantically rich, but ambiguous, and highly dependent on context. This was shown in our judgment task, in which we found ceiling effects for the forced-choice experiment, but relatively low scores for the open question experiment. These findings give rise to some points of discussion.

### Pantomime, not idiosyncratic?

Study 1 showed that there were *default* ways in which individuals depict objects in pantomime: in many cases, most individuals used the same technique to depict a certain object. Furthermore, Study 2 showed that these *defaults* were better understood than objects that did not have a *default* way of depiction. These findings seem to illustrate that the production of pantomime, at least for the items in our dataset, is not fully idiosyncratic. Rather there seem to be certain systematic aspects to how people translate mental representations into pantomimes, which seem to support comprehension.

Although we do not know exactly what processes lead to selection of specific pantomimes, we can speculate as to why these systematic aspects occur. The observation that objects with a *default* technique were better comprehended than objects without a *default* technique suggests that a systematic nature, i.e., everybody does it in the same way, aids comprehensibility. Pantomimes are probably better understood when individuals themselves would make that pantomime similarly. Following the reversed model of Taub (2001a), as we proposed in the introduction, for the comprehension of a pantomime one needs to deduce a scheme from a pantomime. When schemes are shared between interlocutor and pantomimer, this can lead to identification of the same concept. To further explore to what degree pantomimes are conventionalized, it would be interesting to look into whether there are cultural differences in how people depict objects. The study results of Padden et al. (2015) suggest a high consistency even across speakers from different cultures. On the other hand, within this consistency in techniques, there can also be cultural specific information. For instance, Osugi et al. (1999) described that to depict “Fish” deaf and hearing adults used (among other techniques) a handling technique that reflected the specific way in which this fish was caught, e.g., by spear or by hook. Also, it would be interesting to investigate at what age children start to depict objects in a “grown up” way (Overton and Jackson, 1973; Boyatzis and Watson, 1993; Tolar et al., 2008; Weidinger et al., 2014; Masson-Carro et al., 2015), as this could provide further indications regarding how conventions in pantomime arise.

Goldin-Meadow et al. (1996) propose that gesture takes on linguistic properties when it has to carry the “burden” of communication. As Perniss and Vigliocco (2014) discuss, both the need to map linguistic form to experience and the need for an efficient, discriminable signal are central to successful communication. The observed systematic aspects in pantomime in our study may be a first step in this process. In our experiment, as well as in a speaking community, there is no need and not enough “pantomime interaction” for pantomime to become more emblematic or to take on linguistic properties and develop into a more conventionalized gesture system (Hall et al., 2013, 2014; Goldin-Meadow and Brentari, 2015), such as home sign or sign language (Sandler et al., 2005; Brentari et al., 2012).

### Pantomime techniques used to depict distinctive shapes or actions

In Study 1, we found that the *handling* technique was used most frequently, and that this technique was used more often for tools than for non-tools. For animals, the *object* technique was preferred. This result is consistent with previous studies (Padden et al., 2013, 2015; Hwang et al., 2014; Masson-Carro et al., 2016). Study 2 showed that objects depicted by a *handling* technique, and particularly tools, were better understood than objects depicted by other techniques. This finding supports the notion that pantomime is best suited to depict information that is action-based (such as pretending to brush your teeth).

In the introduction of this paper, we discussed that pantomime is probably best suited to depict salient features of objects that are easily translated into the pantomime domain. Unfortunately, little is known on semantics in the gesture domain, and for future research, there is a need for more knowledge on “gesture semantics” (also see Lascarides and Stone, 2009). This could be used to analyze what type of techniques people use in depicting information, but also to find out what type of information is depicted.

We wish to point out that our design, using pictures, may have influenced the “accessibility” of certain features and/or mental representations. Firstly, viewing a picture of an object you could manipulate may have primed the actions associated with using the tool (Ellis and Tucker, 2000; Bub et al., 2003; Glover et al., 2004). Furthermore, pictures obviously visualize the shape of the depicted object, which may partly explain why *shape* gestures were relatively often relied upon as a representation technique. In terms of the model by Gonzalez Rothi et al. (1997), individuals could “copy” the picture into a *shape* gesture. Finally, the pictures used may have influenced the conceptualization of the observed object. For instance, a picture of an “Igloo” with the entrance toward the viewer, may elicit other representations (entering the “Igloo”) when compared to a picture with an entrance facing the side. However, our data show that our participants frequently express information through gesture that is not depicted in the target picture (as for instance showing “pain” for “Cactus,” and “being cold” for “Igloo”). Therefore, it would be interesting for future research to repeat this experiment with spoken and/or written presentation of the targets.

### Pantomime is ambiguous

Study 2 showed that judges were adequate in terms of deriving meaning from pantomimes, as shown by the ceiling effects found in the forced-choice experiment. The meaning conveyed in pantomime, however, seemed ambiguous and unspecific, as people had relatively low Correct scores in the open question experiment. This is in line with results reported by Klima and Bellugi (1979) for the comprehensibility of iconic signs for non-signers. Our findings lend support for our hypothesis that pantomime conveys semantically rich, but imprecise, information, and that its interpretation is highly dependent on context. It is important to note that our experiment probably provides an underestimation of how informative pantomimes can be. Participants who produced the pantomimes knew that the experimenter had to choose between four pictures. In this way the communicative context was comparable between participants. However, participants may have pantomimed less information than when the experimenter would have had no context to choose from. Consequently, in other communicative settings pantomime has the potential to convey even more information than is reflected in our study.

We discussed that, in order to understand pantomimes, individuals need to map the schema they deduce from a pantomime onto their own mental concepts (based on Taub, 2001a). Schematizations probably differ between individuals, resulting in various incorrect answers in our study, such as mismatches (“Couch” instead of “Bed”), but also close alternatives (“Pen” instead of “Pencil”) or category labels (such as “Plant” instead of “Tree”). This illustrates that pantomime, despite being unspecific, did convey semantically rich and useful information. We tried to capture this in the semantic score for the open question experiment. However, as this score is a linguistically based measure, it may not have reflected all the information that was semantically relevant for pantomimes. This again shows a need for more knowledge on gesture semantics.

Handling gestures were better understood than shape gestures. There are various explanations for this. Firstly, handling gestures are probably less ambiguous than shape gestures. As Brentari et al. (2015) point out this particular use of iconicity appears to be grounded in our shared physical experience with the world. For the production of a handling technique one can rely on the underlying cognitive mechanism of perceptuo-motor experience with the objects (Wilson, 2002). Furthermore, for both the production and the comprehension, one can rely on the direct link between the motor action of, for instance, combing one's hair, and the pantomime programs (Hostetter and Alibali, 2008), which is likely to be similar across individuals. Whereas for other pantomimes schematization may differ between individuals, for handling techniques the pantomime would be similar and therefore people were also reasonably good at identifying the exact meaning of these pantomimes. Another explanation could be that the items which were depicted with a handling technique, were often items with which one interacts frequently, such as toothbrush or comb. This is in contrast with items that were not depicted by a handling techniques, such as a rhino or sphinx, with which one does not interact frequently. It is important to note though, that most participants have probably never played an accordion, or tennis, or have been in a wheelchair. Nevertheless, they depict these using handling techniques. Finally, handling gestures naturally give additional information about, for instance, position in relation to the body (a toothbrush is positioned near the mouth and an accordion near one's stomach). This is much less so the case for object and shape gestures for which the position is often, though not always, arbitrary.

Since little is known about how individuals understand pantomimes, we had to come up with a measure to determine comprehensibility of the pantomime using naïve judges. The task used had some advantages as well as disadvantages. First, a difficulty in the construction of the forced-choice task was the selection of distracters. For language there is a range of measures that, depending on the type of task and research question, can be controlled for in experiments: such as word frequency, word length, age of acquisition, phonologic and semantic properties etc., etc. In absence of such measures for pantomime, we chose to use random distracters from the Boston Naming Task, for which at least linguistic factors are well controlled. For some items, this may have led to the use of distracters that were easy to discard as the ceiling effects found in our study indicate that this task was relatively easy to perform. Note though, that it was the aim of these forced-choice questions to investigate whether there was useful information in a pantomime, and not to identify whether a pantomime could be identified correctly without context, as for the latter we used the open-ended questions. Despite the ceiling effects seen on the forced-choice task, it was sensitive enough to show differences in the comprehensibility of tools as compared to non-tools, and objects that were depicted by a *handling* technique as compared to objects depicted by a *shape* technique. Possibly, the forced-choice option will prove to be particularly suited for testing the comprehensibility of pantomimes used by clinical groups, for whom we might not expect ceiling effects. The open question experiment was more sensitive. We should point out though, that this is an indirect scoring system. Possibly, if a judge was unfamiliar with an object (“Yoke” or “Stethoscope,” for instance), this would affect the comprehensibility score of the pantomime for that object. We controlled for this by including nameability as a covariate in our analyses. Furthermore, we have minimized individual impact by using three judges per clip in each experiment.

Finally, we found a minor influence of ability to use both hands on the comprehensibility of pantomimes, in that pantomimes performed by people able to use both hands were slightly better understood than pantomimes performed by people able to use only their left, and non-dominant, hand only. Further research should look into the differences between one- and two handed gestures, taking into account hand preference, to establish whether these factors have an impact on pantomime and gesture production and comprehensibility.

### Pantomime baseline and database

The pantomimes described in this study constitute a pantomime database, which can be accessed online: https://dataverse.nl/dataset.xhtml?persistentId=hdl:10411/QZHO6M. It provides norms for what techniques people use to depict a set of 60 objects and how comprehensible these pantomimes are. As such, it provides a tool which allows clinicians to compare the pantomime behavior in clinical groups to that of healthy speakers. For clinical practice, it is important to know more about pantomime, as the information conveyed by pantomime can benefit the communication of people with language impairments. Based on our findings, we can already draw some general implications for clinical practice. Firstly, between people able to use both hands vs. people restricted to use only their left hand, we found no difference in the type of representation techniques used and only a minor difference in the comprehensibility of the pantomimes they produced. This indicates that people able to use only one hand, as is often the case in people with aphasia (Brust et al., 1976), do not necessarily have to be excluded from pantomime therapy. Furthermore, we saw that handling techniques were used frequently, particularly for depicting tools, and that these were best understood. In pantomime therapy, it may be beneficial to start with these “easy” items. The general effectiveness of such therapies should be determined in future research. Finally, we found that the information conveyed in pantomime is ambiguous. For clinical applications, this means that interlocutors need to take an active role in communication by checking and disambiguating the information conveyed in a pantomime, by asking questions, for instance. For a more detailed discussion of clinical implications for pantomime use by people with aphasia, see van Nispen et al. (2016).

## Conclusion

Similar techniques were used across individuals to depict objects in pantomime. This showed that pantomime is not fully idiosyncratic. As pantomime is based on people's mental representation of objects, the observed systematic aspects seemed to be a result of intrinsically similar mental representations and similar strategies to translate these, using iconicity, into pantomime. The meaning of pantomimes is semantically rich, albeit ambiguous and therefore highly context dependent. Interpretation of a pantomime is not based on conventions, but depends on the interpretation of the interlocutor and the context is which it is produced. Individuals probably rely on their own schematization of a concept, and overlap in schemes between pantomimer and interlocutor may lead to mutual understanding. This seemed most easily achieved for *handling* techniques, often used for depicting tools, which were better understood than the other techniques. This is probably because of the motor program used to perform these actions, which is shared between pantomimer and interlocutor. Our study has resulted in a pantomime database which is available online: https://dataverse.nl/dataset.xhtml?persistentId=hdl:10411/QZHO6M. It provides pantomime norms for 60 well-documented objects from the Boston Naming Task that could be used for comparison with clinical groups.

## Ethics statement

The present study obtained ethical approval from the Medical Ethical Review Committee of the Erasmus University Medical Centre, Rotterdam. The present study only reports on the data of healthy individuals. Human participants gave informed consent before taking part in the study.

## Author contributions

KvN was the main author of this paper. EK and WMEvdSK were highly involved in designing the studies, analyzing the results and revising the text of the manuscript.

### Conflict of interest statement

The authors declare that the research was conducted in the absence of any commercial or financial relationships that could be construed as a potential conflict of interest.
