# Supplementary material for: Production and Comprehension of Pantomimes Used to Depict Objects
Source: Front Psychol. 2017 Jul 11;8:1095. doi: 10.3389/fpsyg.2017.01095 (PMC5504161; doi:10.3389/fpsyg.2017.01095)
Supplement: Supplementary file 1 [file DataSheet1.docx]

1. Appendix

| **Table A1** Description of the Semantic score open (based on van Loon-Vervoorn, Stumpel, & de Vries, 1996) | | |
| --- | --- | --- |
| Score | Description | Example: Object [01] Bed |
| 3 | Correct word or synonym | Bed, single bed |
| 2 | -Incorrect response, but semantically appropriate  -Description of the word  -Coordinate  -Second part of a composite | Cradle  To sleep on  Furniture  n..a (e.g. horse for seahorse) |
| 1 | Incorrect response but semantically slightly suitable | sleeping |
| 0 | All other incorrect responses | Square, box |
